# Sharp Curvature of Premolar Resulting in External Apical Root Resorption of the Neighbouring Molar

**DOI:** 10.1155/2011/560684

**Published:** 2011-07-03

**Authors:** Özgür İlke Atasoy Ulusoy

**Affiliations:** Department of Restorative Dentistry and Endodontics, Faculty of Dentistry, Gazi University, 82 Street, Emek 06510 Ankara, Turkey

## Abstract

This case report describes an external apical root resorption resulted from the unusual root morphology of the neighbouring tooth. A 28-year-old female was referred to the department of endodontics with a complaint of intense pain in her maxillary second premolar tooth. The clinical and radiographical evaluation revealed an external apical resorption in the mesial root of the maxillary first molar, which shows close proximity to the severely curved root of the premolar. A successful root canal treatment of the premolar was performed using anticurvature filing method. However, molar tooth received no curative treatment. One-year followup of the apical external resorption did not show any progression. External apical root resorption affecting single permanent tooth may be induced from the pressure exerted during the eruption of the adjacent tooth with unusual root morphology. The preferred approach for the management of such apical resorption cases includes long-term observation and no curative treatment.

## 1. Introduction

The initiation and progression of root resorption is usually pathological in permanent dentition and attributed to several factors including mechanical trauma, inflammation, orthodontic tooth movement, periodontal problems, neoplastic lesions such as cysts and tumours, impacted teeth and systemic diseases [1–3]. The pathological root resorption can be broadly classified as internal or external including subdivisions [1]. External root resorption, affecting multiple or single teeth, is commonly located in apical and cervical thirds of the root canal system [2, 4, 5].

The shape of the root canal system may show variations including severe curvatures and complicate endodontic treatment. Severely curved roots showing close proximity to the anatomical structures such as maxillary sinus, inferior alveolar nerve, or the neighbouring tooth may also result in complications like inflammation, mechanical trauma, or resorption. 

This paper describes the endodontic treatment of a maxillary premolar with a severely curved canal in association with external apical resorption in the mesial root of the neighbouring molar. Although other cases of root resorption caused by various reasons have been described in the literature [2, 5], there is no report representing a resorption case induced from the sharp curvature of the adjacent tooth.

## 2. Case Presentation

**Figure 1:**
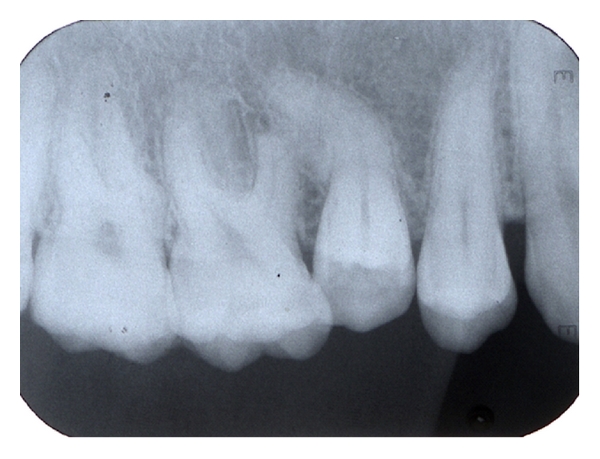
Initial periapical radiograph showing external apical root resorption resulted from the severely curved root of the adjacent premolar.

**Figure 2:**
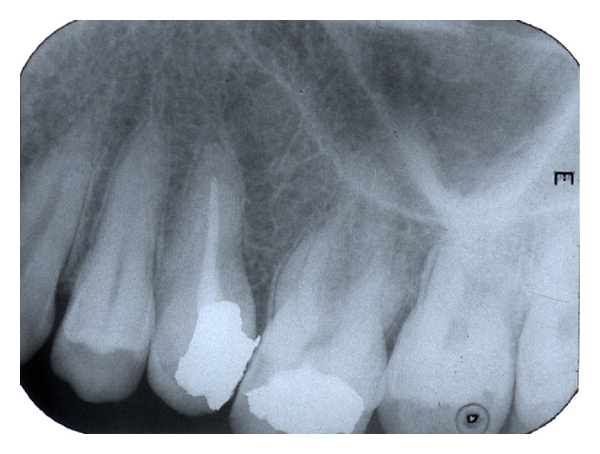
Periapical radiograph taken from the symmetrical region of the maxilla shows no unusual morphology of the teeth.

A 28-year-old female was referred to the Department of Restorative Dentistry and Endodontics of Gazi University Dental Faculty with a complaint of spontaneous pain. Clinical examination showed a fully dentate patient with deficient oral hygiene. Tooth 15 had a carious lesion which was verified by the radiographic examination. The clinical crown of 15 was tipped in the palatine direction and positioned 2 mm below the occlusal plane. Radiographic examination also revealed a sharp curvature located in the middle third of the root canal (Figure 1). The apical third of the root 15 seems to have direct contact with the mesial root of 16 which was shortened severely (Figure 1). The remaining teeth showed no unusual tooth morphology. The mobility of all teeth was within the physiological range. A periapical radiograph taken from the symmetrical region in the maxilla exhibited no abnormality in the root shape and morphology of the teeth 25 and 26 (Figure 2). The medical history of the patient revealed no systemic disorder, an endocrine or a metabolic disease. She had no history of previous orthodontic treatment.

On the basis of the clinical and radiographical evaluation, the definitive diagnose of tooth 16 was external apical root resorption related to excessive pressure formed by unusual root morphology of the adjacent tooth. As this phenomenon did not mediate within the pulp chamber and the tooth-responded electric pulp testing, endodontic treatment of 16 was not indicated to arrest this type of external apical resorption. The tooth 15 was diagnosed as irreversible pulpitis, and endodontic treatment was initiated. The tooth was anesthetized with 2% articaine with epinephrine 1 : 200000 (Ultracaine DS, Hoechst Marion Roussel, Germany), and endodontic access cavity was prepared under rubber-dam isolation.

Intraoral examination confirmed one oval shaped root canal. The working length was determined with an electronic apex locator (Root ZX, J. Morita Corp., Tokyo, Japan) and controlled with a periapical radiograph. The root canal was biomechanically prepared with H and K type files using an anticurvature step-back technique to a master apical file of 35. During instrumentation, the root canals were irrigated with 2 mL of 2.5% NaOCl (Wizard, Rehber Chemistry, Istanbul, Turkey). Final irrigation was performed with 2 mL of 15% EDTA (Wizard, Rehber Chemistry, Istanbul, Turkey), followed by a wash of 2.5% NaOCl. 

**Figure 3:**
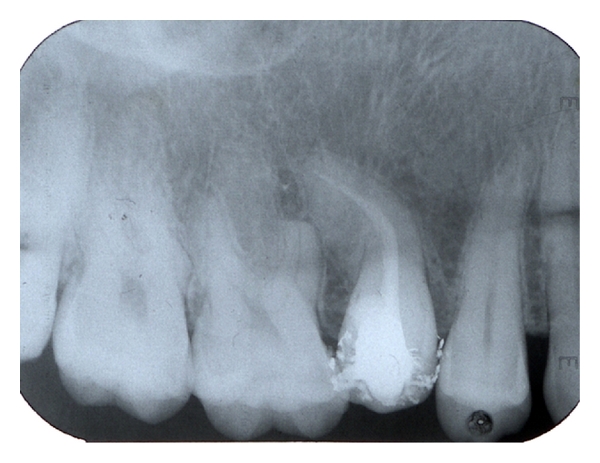
Postoperative radiograph following root canal treatment of the premolar.

Calcium hydroxide-based intracanal dressing was applied in the instrumented root canal with a lentulo spiral. Access cavity was sealed with a temporary filling material (Cavit, ESPE, Seefeld, Germany). One week later, the root canal was obturated by the cold lateral condensation technique with standardized gutta-percha points and AH plus sealer (Dentsply de Trey, Konstanz, Germany). Another periapical radiograph was exposed to check the quality of obturation (Figure 3). The cavity was restored with amalgam.

**Figure 4:**
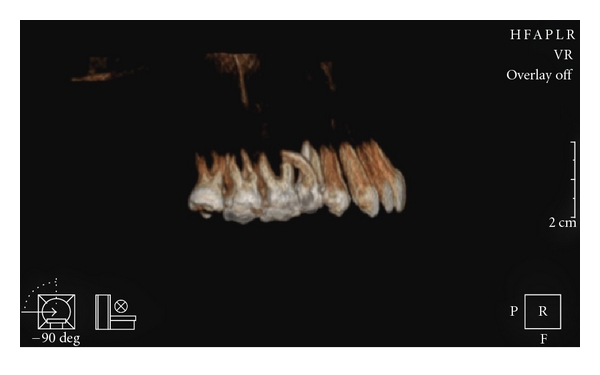
Reconstructed cone-beam computerized tomography image showing right side of the maxilla.

**Figure 5:**
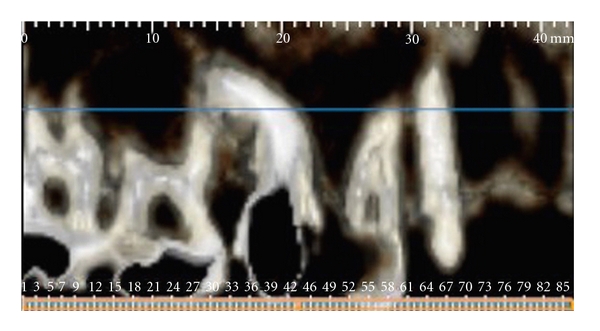
Cone-beam computerized tomography image showing close association between the roots of the maxillary second premolar and first molar.

The patient was instructed to return for preservation radiographic exams, first, within 3 months; and finally 1 year after the completion of the endodontic treatment. After 1 year, clinical examination detected a small caries lesion in the occlusal surface of the tooth 16, which was restored with amalgam restoration. At this time, a cone-beam computerized tomography (ILUMA Cone Beam CT, IMTEC Imaging, Ardmore, Okla) of the maxilla was performed with a tube voltage of 120 kVp and tube current of 3.8 mA, for better definition and visualization of the root lengths and morphology (Figures 4 and 5). It was confirmed that endodontic treatment of the premolar had been successful, and there was no progression of the external root resorption in the neighbouring molar tooth.

## 3. Discussion

The root canal morphology of teeth is often quite complex and highly variable [6]. Vertucci [7] reported that maxillary premolars show maximum anatomic variations such as “S” or bayonet-shaped root canals. In the presence of curvatures, it is very difficult to achieve complete shaping and cleaning the root canal system [8]. In the current case, we did not use any tapered files or rotary instruments to avoid thinning and perforating the root canal wall due to excessive flaring. Instead, we performed anticurvature filing using step-back technique which allows a controlled and directed canal preparation and reduces the risk of perforation through the furcal or curved root surfaces [9, 10]. Knowledge of the unusual root canal morphology, careful interpretation of angled radiographs, proper precautions, and planning are essential prerequisites for a successful treatment outcome [8].

Root resorption is the destruction, and subsequent loss of the root structure. Although a permanent tooth is located in conjunction with alveolar bone surrounded by multinucleated cells, it is not affected by any of them and resorption under normal conditions [11]. External root resorption is a pathological process which tends to occur following a wide range of mechanical or chemical stimuli [12]. Reports of systemic disease-induced or idiopathic external apical root resorption affecting the multiple permanent teeth are existing in the literature, although they are rare [2, 13]. External apical root resorption in association with a single tooth is commonly resulted from inflammation [14]. However, there is no such an interesting case as the present one which involves external apical resorption caused from the anatomic variation of the neighbouring tooth. 

In this case, the root resorption of the molar may be due to the osteoclast differentiation related to the pressure exerted during the eruption of the permanent maxillary second premolar with a severely curved root. It may resemble the external resorptions resulted from the pressure due to the ectopic, impacted teeth or a pathologic lesion like cysts or tumours erupting in the path of the root [15]. Radiographs of the patient before the original referral were unfortunately not available for a conclusive decision on the real reason of the pathology. Therefore, cone-beam computerized tomography was carried out to confirm the unusual morphology and proximity of the roots.

 In the present case, conventional root canal treatment was performed on the severely curved root of the maxillary second premolar. Whereas, no curative treatment was undertaken for external apical root resorption of the molar tooth. Root canal therapy may be beneficial in the external and internal resorptions that are arisen from inflammation. However, endodontic treatment is not indicated in the replacement and pressure resorption cases [1]. The current management for such resorption cases is based on minimal intervention and long-term observation [2].

In conclusion, knowledge of the aetiology and radiographic characteristics is essential for the treatment of external apical root resorption. Most of the affected teeth are asymptomatic as in this case, and they are discovered in the radiographs taken incidentally. The current management for external root resorption includes minimal intervention and long-term monitoring in the absence of clinical signs and symptoms of pulpal inflammation.
